# Complications of chronic total occlusion percutaneous coronary intervention

**DOI:** 10.1007/s12471-020-01502-1

**Published:** 2020-10-27

**Authors:** J. Karacsonyi, E. Vemmou, I. D. Nikolakopoulos, I. Ungi, B. V. Rangan, E. S. Brilakis

**Affiliations:** 1 Minneapolis Heart Institute and Minneapolis Heart Institute Foundation Abbott Northwestern Hospital, Minneapolis, MN USA; 2 Division of Invasive Cardiology, Second Department of Internal Medicine and Cardiology Centre, University of Szeged, Szeged, Hungary

**Keywords:** Complication, Percutaneous coronary intervention, Chronic total occlusion

## Abstract

Chronic total occlusion percutaneous coronary interventions can be highly complex and are associated with an increased risk of complications, such as perforation, acute vessel closure (which can lead to rapid haemodynamic compromise if it involves the donor vessel), and equipment loss or entrapment. Awareness of the potential complications and meticulous attention to equipment position and patient monitoring can help minimise the risk of complications and allow prompt treatment should they occur.

## Introduction

Despite its clinical benefits [1, 2] chronic total occlusion (CTO) percutaneous coronary intervention (PCI) is associated with higher complication rates than PCI of non-occlusive lesions [3]. CTO PCI complications include death, acute myocardial infarction, stroke, the need for repeat PCI, emergency coronary artery bypass graft surgery, tamponade requiring pericardiocentesis or surgery, acute vessel closure (which can be a catastrophic complication if it involves the CTO donor vessel), coronary dissection, aorto-ostial dissection, thrombus, embolisation of thrombus, plaque or air, side branch occlusion, spasm, pseudolesion formation, intramural haematoma, perforation, equipment entrapment/loss, hypotension, arrhythmias, vascular access complications and bleeding, contrast-induced acute kidney injury, and radiation skin injury [4]. The complications of CTO PCI can be classified as acute and long-term based on timing. CTO PCI complications can also be classified according to location into cardiac and non-cardiac complications. Cardiac complications can be further divided into coronary and non-coronary (Tab. 1). Each complication has a different mechanism and underlying causes. A score has been developed for estimating the risk of periprocedural complications using the following three parameters: patient age >65 years, +3 points; lesion length ≥23 mm, +2 points; and use of the retrograde approach, +1 point [5].

**Table 1:** Types of complications during chronic total occlusion (*CTO*) percutaneous coronary interventions (*PCI*)

Acute complications of CTO PCI	
Cardiac complications	Non-cardiac complications
*Coronary complications*	
Acute vessel closure	– Vascular access complication
– Donor vessel injury	– Contrast-related nephropathy
– Occlusion of collaterals	– Allergies
– (Aorto)coronary dissection	– Radiation skin injury
– Dissection of distal vessel	– Thromboembolic complications
– Side branch occlusion	– Stroke
– Thrombus	
– Spasm	
– Pseudolesion formation	
– Subintimal stent deployment	
– Embolisation: *–* thrombus, – plaque, – air	
Perforation:	
– Large vessel	
– Collateral	
– Distal vessel	
Equipment entrapment/loss	
*Non-coronary complications*	
– Hypotension	
– Myocardial infarction	
– Arrythmias	
– Death	
– Intramural haematoma	
– Tamponade	

## Donor vessel injury

Donor vessel injury requires immediate identification and management, as it can lead to extensive ischaemia and haemodynamic decompensation [6]. In a meta-analysis of retrograde CTO PCIs, donor vessel dissection occurred in 2% of treated CTOs (95% confidence interval: 0.9–4.5%) [7].

**Fig. 1:**
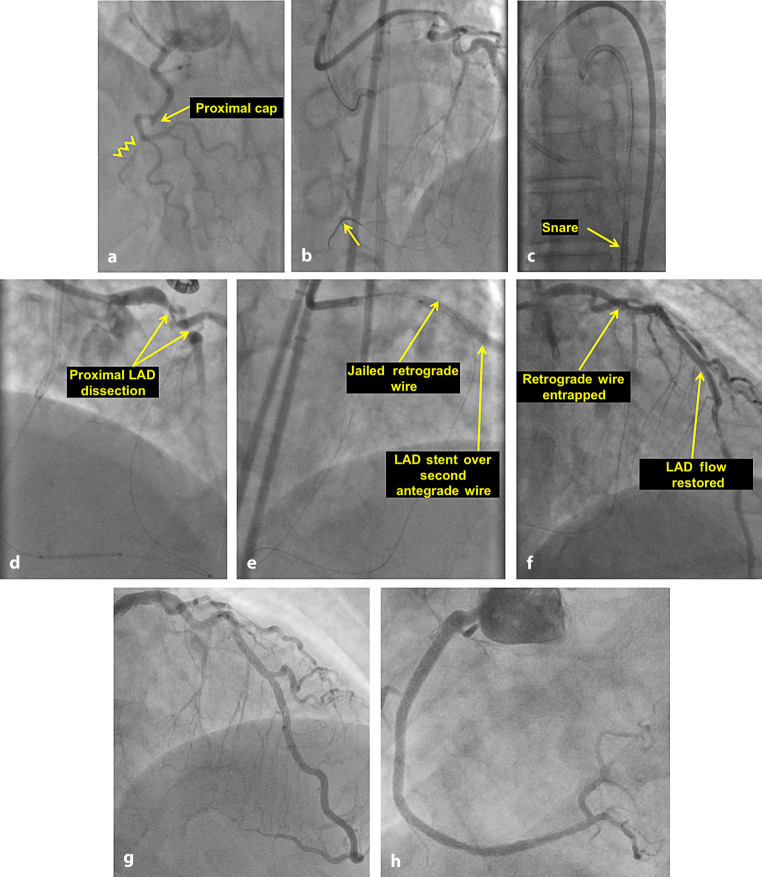
Example of donor vessel dissection during retrograde chronic total occlusion (CTO) percutaneous coronary intervention (PCI). PCI of a right coronary artery (RCA) CTO (**a**). After a failed antegrade crossing attempt, retrograde crossing was performed (**b**) and the retrograde guidewire was externalised (**c**). During RCA stenting over the externalised guidewire (**d**), the patient developed severe chest pain and hypotension due to proximal left anterior descending artery (*LAD*) dissection (**d**). The *LAD* was immediately stented (**e**) with restoration of antegrade flow and stabilisation of the patient (**f**, **g**). After removal of the entrapped retrograde guidewire and stenting of the RCA an excellent final angiographic result was achieved (**h**). Reproduced with permission from [6]. Online case with video is available on https://www.ctomanual.org/Case 22

Donor vessel injury may be due to dissection caused by deep catheter engagement, for example during equipment withdrawal or during wire externalisation when the operator pulls the retrograde wire forcefully (Fig. 1). Flow in the donor vessel can also be compromised due to catheter or vessel thrombosis, which may be due to long procedures with decreasing activated clotting time (ACT), blood stasis, especially in diseased donor vessel and failure to regularly clear the guide catheter, particularly after trapping [6].

To prevent this complication paying close attention to the position of the guide catheters and to the pressure waveforms is essential, especially during externalisation. Side-hole guide catheters should not be used in the donor vessel, as they can mask pressure dampening which can lead to ischaemia. The ACT should be kept above 300 s (for antegrade procedures) and 350 s (for retrograde procedures), checking it every 20–30 min throughout the procedure. Moreover, retrograde CTO PCI should not be performed through significantly diseased donor vessels to minimise the risk of ischaemia: donor vessel lesions should be treated first prior to advancing microcatheters and attempting retrograde crossing. A ‘safety’ guidewire should be placed in the donor vessel to facilitate treatment should donor vessel occlusion occur [6].

Donor vessel injury should in most cases lead to discontinuation of the CTO PCI attempts, focusing all efforts on restoring the patency of the donor vessel. Haemodynamic support may be required in the case of haemodynamic compromise. Dissections are treated with stenting, ideally over the safety guidewire after removal of the externalised guidewire. Thrombotic occlusion is treated by thrombectomy and possibly the administration of intravenous antiplatelet medications [6].

## Perforation

Coronary perforation is one of the most feared complications of CTO PCI [8]. In a recently published analysis of 1811 cases from five European centres it occurred in 5.5% of the CTO PCIs, with more than half of these cases requiring management and 20% resulting in tamponade. The following characteristics were found to be independently associated with coronary perforation: older age, occlusion length >20 mm, rotational atherectomy, antegrade dissection/re-entry, and use of the retrograde approach [9]. In another multicentre US registry analysing 2097 CTO PCIs performed in 2049 patients, the incidence of perforation was 4.1%, with 14% of the patients developing tamponade requiring pericardiocentesis. In this study, age, previous PCI, right coronary artery target CTO, blunt or no stump, use of antegrade dissection re-entry, and the retrograde approach were associated with perforation [10]. The retrograde approach has been associated with a higher risk of perforation, although in recent analyses many of the perforations observed during retrograde CTO PCI were due to antegrade crossing attempts [11, 12].

**Fig. 2:**
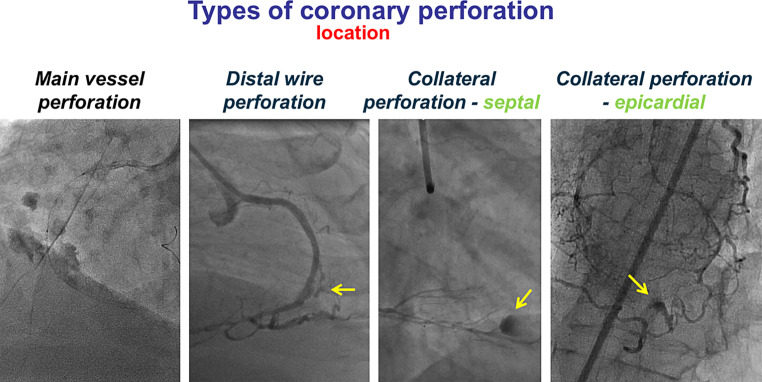
Types of coronary perforation based on location. Reproduced with permission from [33]

Coronary artery perforations have traditionally been classified based on severity (Ellis classification). Class 1: a crater extending outside the lumen only in the absence of linear staining angiographically suggestive of dissection. Class 2: Pericardial or myocardial blush without a larger than 1 mm exit hole. Class 3: Frank streaming of contrast through a ≥ 1-mm exit hole. Class 3‑cavity spilling: Perforation into an anatomic cavity chamber, such as the coronary sinus, the right ventricle, etc. [13]. The location of the perforation is also critically important, as it has important implications regarding management [6]. There are three main perforation locations: (a) large vessel perforation, (b) distal vessel perforation, and (c) collateral vessel perforation, in either a septal or an epicardial collateral (Fig. 2; [14–16]). Large vessel perforations are more common than distal vessel perforations [17].

**Fig. 3:**
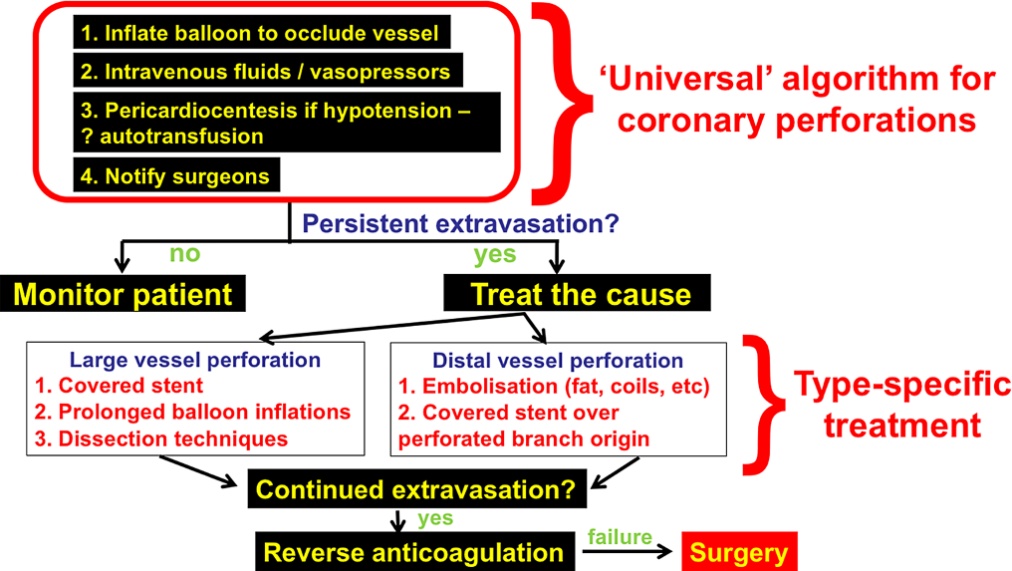
Coronary perforation management algorithm

The risk of perforation can be minimised by meticulous attention to equipment during CTO crossing attempts. Guidewire position within the vessel ‘architecture’ should be confirmed before advancing microcatheters and other equipment. Coronary perforation may lead to cardiac tamponade, myocardial infarction, rapid haemodynamic collapse, and death [18]. The first step in managing a perforation is to inflate a balloon proximal to or at the perforation to stop bleeding into the pericardium (Fig. 3). Large vessel perforations are usually treated with covered stent implantation, although dissection/re-entry techniques have also been successfully used in some cases [19]. Distal vessel perforations are treated with embolisation, usually with fat or coils. Covered stents and/or coils can often be delivered through a single guide catheter, especially if 8‑French guides are used [20]. Alternatively the dual guide catheter technique can be employed with one guide catheter used for delivering a balloon to achieve haemostasis and the second guide catheter for covered stent delivery. Availability of 0.014-inch coils can facilitate delivery through standard microcatheters, as larger 0.018-inch coils require larger microcatheters [such as the Progreat (Terumo, Tokyo, Japan) or Renegade (Boston Scientific, Marlborough, MA, USA)] or use of the Finecross microcatheter (Terumo). Storage of perforation management equipment (covered stents, coils, pericardiocentesis kit) in a CTO or complex PCI cart can expedite treatment [21].

## Side branch occlusion

Occlusion of the side branches can develop, especially when subintimal dissection/re-entry strategies are applied in CTO PCI, and has been associated with a higher risk of post-PCI myocardial infarction [22, 23]. Extensive dissection/re-entry strategies, such as the subintimal tracking and re-entry (STAR) technique, are associated with high rates of restenosis and reocclusion likely due to side branch occlusion and decreased outflow [24]. The extent of dissection should, therefore, be limited [23, 25]. Moreover, side branch wiring before stenting can help prevent occlusion and can be facilitated by use of dual lumen microcatheters, such as the Twin Pass (Teleflex, Wayne, PA, USA), Crusade (Kaneka, Tokyo, Japan), NHancer Rx (IMDS, Roden, The Netherlands) or Sasuke (Asahi Intecc Co., Seto, Japan). In some cases a retrograde crossing strategy can be applied to preserve side branches [6, 26]. Intravascular imaging, particularly intravascular ultrasound, can help to determine the mechanism of side branch loss and also facilitate re-opening [6].

## Equipment loss or entrapment

This complication is rare but potentially could be life-threatening depending on the device and location of the entrapment or loss. Stents are the most commonly embolised devices with an estimated incidence of 0.32% [27]. Equipment delivery can be challenging during CTO PCI, especially through tortuosity and calcification [28]. Retrograde equipment delivery should be avoided [29] as well as excessive guidewire and microcatheter rotation and aggressive Rotablator burr advancement [30, 31]. Use of smaller burrs, advancement of the burr using a pecking motion and avoidance of sudden decelerations is advised [32]. Before attempting stent delivery the target lesion should be carefully prepared with balloon angioplasty and atherectomy if necessary. Checking the transmission of torque to the guidewire tip, and alternating clockwise and counter-clockwise microcatheter rotation, can help minimise the risk of equipment loss/entrapment.

Should equipment loss or entrapment occur, the first decision is whether to attempt retrieval or deploy/crush the equipment against the vessel wall. For stent loss in coronary segments that are unlikely to be significantly affected by the stenting, deployment is often the preferred strategy, as stent retrieval attempts may result in distal stent embolisation or target vessel injury [27]. If crushing is the best option intravascular imaging should be performed to ensure an optimal PCI result [6]. If retrieval is attempted, various snares, most commonly three-loop snares, are most often used.

## Conclusions

CTO PCI can lead to potentially life-threatening complications. Awareness of such complications, meticulous using techniques to minimise risk, using and prompt recognition and treatment can optimise CTO PCI outcomes.
